# Temporal dynamics of the bat wing transcriptome: Insight into gene-expression changes that enable protection against pathogen

**DOI:** 10.1080/21505594.2022.2156185

**Published:** 2023-01-04

**Authors:** Aoqiang Li, Haixia Leng, Zhongle Li, Longru Jin, Keping Sun, Jiang Feng

**Affiliations:** a Jilin Provincial Key Laboratory of Animal Resource Conservation and Utilization, Northeast Normal University, Changchun, China; b School of Life Sciences, Central China Normal University, Wuhan, China; c College of Life Science, Jilin Agricultural University, Changchun, China

**Keywords:** Wing transcriptome, immune defence, *Pseudogymnoascus destructans*, *Rhinolophus ferrumequinum*, hibernation

## Abstract

Skin acts as a mechanical barrier between the body and its surrounding environment and plays an important role in resistance to pathogens. However, we still know little regarding skin responses to physiological changes, particularly with regard to responses against potential pathogens. We herein executed RNA-seq on the wing of the *Rhinolophus ferrumequinum* to assess gene-expression variations at four physiological stages: pre-hibernation, hibernation (early-hibernation and late-hibernation), and post-hibernation, as well as the gene-expression patterns of infected and uninfected bats with the *Pseudogymnoascus destructans* (*Pd*). Our results showed that a greater number of differentially expressed genes between the more disparate physiological stages. Functional enrichment analysis showed that the down-regulated response pathways in hibernating bats included phosphorus metabolism and immune response, indicating metabolic suppression and decreased whole immune function. We also found up-regulated genes in post-hibernating bats that included C-type lectin receptor signalling, Toll-like receptor signalling pathway, and cell adhesion, suggesting that the immune response and skin integrity of the wing were improved after bats emerged from their hibernation and that this facilitated clearing *Pd* from the integument. Additionally, we found that the genes involved in cytokine or chemokine activity were up-regulated in late-hibernation compared to early-hibernation and that *FOSB* regulation of immune cell activation was differentially expressed in bats infected with *Pd* during late-hibernation, implying that the host’s innate immune function was enhanced during late-hibernation so as to resist pathogenic infection. Our findings highlight the concept that maintenance of intrinsic immunity provides protection against pathogenic infections in highly resistant bats.

## Introduction

Hibernation is a seasonal physiological adaptation that allows some mammals to survive in harsh winter circumstances with limited food availability [1]. During hibernation, much of this time is spent in torpor, a physiological stage characterized by inactivity, attenuated body temperature, reduction in heart and metabolic rates, and immune suppression [2–4]. While the molecular and genetic bases of hibernation physiology in mammals have been partially evaluated by analysing the differential gene expression in some species such as bears, ground squirrels, primates, and bats [5–8], the majority of extant studies focused on determining the differentially expressed genes in the brain, heart, and liver – organs involved in carbohydrate and lipid metabolism, detoxification, and molecular transport [9–11].

Animal skin, which is the tissue that serves as a portal between the body and external environment, acts as a mechanical barrier in protecting against pathogens. An abundance of evidence now suggests that the skin is actually an active immune organ [12–15]. Additionally, due to its direct contact with the external surroundings, skin is continuously exposed to large numbers of pathogenic. Thus, animal skin may undergo marked physiological changes that cause alterations in gene expression to combat potential pathogens, such as local inflammation response and the production of cytokines, especially during hibernation when the host’s systemic immune function is inhibited. Therefore, exploring gene expression in skin during hibernation is essential to understand how the host copes with potential pathogens and also contributes to understanding the skin’s adaptation to environmental changes. However, little is known regarding changes in skin gene expression during hibernation in mammals, particularly with respect to bat species that are at risk of population extinction due to *Pseudogymnoascus destructans* (*Pd*) infection.

The emerging infectious disease known as white-nose syndrome (WNS) (also called white-nose disease [WND]) is caused by *Pd* (previously referred to as *Geomyces destructans*), and WNS has precipitated a catastrophic decline in the bat fauna of North America [16–18]. *Pd* invades the wing tissues, forming characteristic cup-shaped erosions and ulcerations [19]. Bats awaking from hibernation may also undergo an inflammatory immune-reconstitution syndrome and an acute inflammatory response to cope with *Pd* infection, potentially leading to their mortality [20]. Intriguingly, bat species are seemingly unequally affected by *Pd* [21–23]. For example, many North American bats have been severely affected, while Chinese bats were also infected with *Pd* without any mortality. Variations in skin responses to *Pd* may play a role in the inherently different susceptibilities to *Pd* between North American and Chinese bat faunas. This variety of responses is similar to that reflected in some previous studies on amphibians where those resistant to *Batrachochytrium dendrobatidis* exhibited up-regulated genes involved in pathways associated with maintaining structural integrity of skin (e.g. cell adhesion and epidermis development), whereas susceptible amphibians did not [24,25]. Although limited data on skin responses to *Pd* infection [26–30] as well as on differences in the chemical composition of bat epidermis [31] in susceptible and tolerant bat species have been reported, there are no extant studies on bats that manifest high resistance to *Pd* in endemic regions. We posit that studying the mechanisms by which highly resistant bats cope with *Pd* infection would facilitate revealing the adaptive evolutionary processes associated with the occurrence or reduction of fungal infectious-disease severity. This is critical to our understanding of pathogen virulence and bat survival in natural immune states.

In the present study, we used next-generation RNA sequencing on wing tissues from wild greater horseshoe bats (*Rhinolophus ferrumequinum*) that exhibit high resistance to *Pd* infection so as to provide a more complete picture of the changes in wing gene expression in hibernating and active bats. We assume that *R. ferrumequinum* is resistant rather than tolerant due to the fact that resistance refers to host defences that reduce pathogen growth, whereas tolerance refers to host defences that reduce damage experienced by the host without reducing pathogen growth [32]. A previous study showed that although Chinese *R. ferrumequinum* was similar in colony size and temperatures in hibernation to North American bats, it manifested very low infection intensity and *Pd* load in China, suggesting host resistance to pathogen [21]. Herein, our aims were to 1) to explore the temporal changes in the bat wing transcriptome and to seek critical responses for coping with potential pathogens, and 2) to compare skin responses to *Pd* infection during late-hibernation.

## Materials and Methods

### Sample collection

We collected 30 *R. ferrumequinum* adults over a longitudinal time-course that spanned four physiological stages between October of 2020 and May of 2021 from a hibernaculum in Jilin Province of Northeast China where we had detected *Pd* in previous studies [22,33]. The four physiological stages were pre-hibernation (five individuals in October), early-hibernation (five individuals in December), late-hibernation (12 individuals in April), and post-hibernation (eight individuals in May). We captured active bats with a net (8 m × 3 m) when they flew out of the cave at night, and removed them from the net using sterile latex gloves. After 2 h, we untangled the nets and brought them back to the laboratory to prevent interfering with bat predation. Since only *R. ferrumequinum* lives in this cave, no other bat species were caught during sampling. We captured hibernating bats from their roosting locations. We swabbed each individual bat five times along its forearm and muzzle using sterile polyester swabs for *Pd* detection and punched 5-mm biopsy samples from areas of the right and left plagiopatagium (the membranous area between the last digit and the hindlimbs) of the wing. We then stored the samples in RNase-free tubes containing 500 μL of RNAlater (TIANGEN, Beijing, China) and flash-froze the tubes in liquid nitrogen for RNA extraction. Weights were measured and recorded to estimate the relative physical condition of each individual [34], and bats were then released immediately after sampling.

### Pseudogymnoascus destructans *test*

We extracted DNA from each fungal sample according to DNeasy blood and tissue extraction kits (Qiagen, Hilden, Germany) using the manufacturer’s standard extraction protocol [22,33]. We then employed qPCR to determine the presence of *Pd* [35]. Fungal load was calculated according to the following formula: fungal load = log (10^((Ct − 22.04942)/-3.34789)) [36].

## RNA extraction and transcriptome sequencing

We extracted RNA from each punch biopsy sample of bat tissue based on the TRIzol method (TIANGEN, Beijing, China). We quantified and assessed RNA integrity using the Bioanalyzer 2100 System (Agilent Technologies, CA, USA). RIN values greater than 8.0 were confirmed for all samples in this study. RNA sequencing was subsequently executed on the Illumina HiSeq 4000 platform with paired-end 150-bp nucleotides by the Beijing Allwegene Technology Co. Ltd, China.

## Mapping analysis and quantification of gene-expression levels

After obtaining raw data, quality control was performed using Trimmomatic v 0.33 according to the following criteria: 1) removing reads with adapter sequences; 2) filtering out reads with uncertain bases (N) greater than 10%; 3) filtering out reads with low quality bases (Q < 20) greater than 50% [37]. We then mapped the clean reads to the reference genome of *R. ferrumequinum* from Ensembl 100 using STAR v 2.5.2b [38]. Only reads with a perfect match or with one mismatch were further analysed and annotated based on the bat reference genome. We exploited HTSeq v 0.5.4 p3 to count the read numbers mapped to each gene [39] and calculated the values for fragments per kilobase of transcript, per million mapped reads (FPKM) using RSEM v1.3.1 to estimate gene-expression levels [40,41].

## Differential expression analysis

DESeq2 v1.32.0 was employed to detect differentially expressed genes (DEGs) for each sample pair [42], and resulted in six sets of DEGs, i.e. early- vs. pre-hibernation, late- vs. early-hibernation, post- vs. late-hibernation, late- vs. pre-hibernation, post- vs. pre-hibernation, and post- vs. early-hibernation. To explore whether gene expression differed between wing tissues of infected and uninfected *Pd* bats, we used DESeq 2 to screen for DEGs between the two groups during late-hibernation, and *P* values were corrected based on the Benjamini and Hochberg method [43]. In this study, genes with an adjusted *P* value <0.05 and |log2 fold-change| > 1 were considered to be differentially expressed. In each pairwise comparison, we defined specific up- and down-regulated genes, with up-regulated genes designating expression at higher levels in the former physiological stage than in the latter, while down-regulated genes reflected attenuated expression in the former physiological stage relative to the latter.

To visualize gene-expression patterns across all 30 samples of uninfected bats and bats infected with *Pd*, PCA and hierarchical clustering with Pearson’s correlation were performed within R v. 4.1.0 using *dudi.pca()* and *hclust()* functions [44]. ANOSIM (Analysis of Similarity) based on Euclidean distance was implemented using the *anosim()* function in the vegan package to assess differences between groups. To determine the biological function of DEGs identified in each comparison, functional annotation was carried out by GO and KEGG pathway analyses using the OmicShare tool (https://www.omicshare.com) [45,46]. We achieved multiple-test correction using the Benjamini-Hochberg approach and determined significance with an adjusted *P* value of <0.05.

## Results

### Pd-infection status of R. ferrumequinum

While we detected no *Pd* infection in the bats collected from pre-, early-, and post-hibernation groups, for bats collected during late-hibernation, we detected six bats infected with *Pd*, with an average fungal load was −5.38, ranging from −6.24 to −4.58 per individual (Ct values ranged from 37.38 to 42.94). We also ascertained that infected and uninfected *Pd* bats possessed a similar weight, suggesting their similar physical condition (t = 0.81, *P* = 0.24).

## Transcriptomic sequencing and mapping

We obtained 1,378.52 million raw reads from the 30 samples, with an average of 45.95 million (range, 39.11–54.39 million/sample) raw reads per sample (Table S1). After quality control, we obtained a total of 1,355.03 million clean reads, with approximately 43.70, 49.51, 43.62, and 45.69 million clean reads retrieved from pre-, early-, late-, and post-hibernation groups, respectively. Of these, 38.34, 43.74, 38.35, and 39.59 million clean reads were mapped to the *R. ferrumequinum* reference genome, with average alignments of clean reads to the reference genome of 87.73%, 88.35%, 87.82%, and 86.65%, respectively.

## Differential expression at four physiological stages

**Figure 1.**
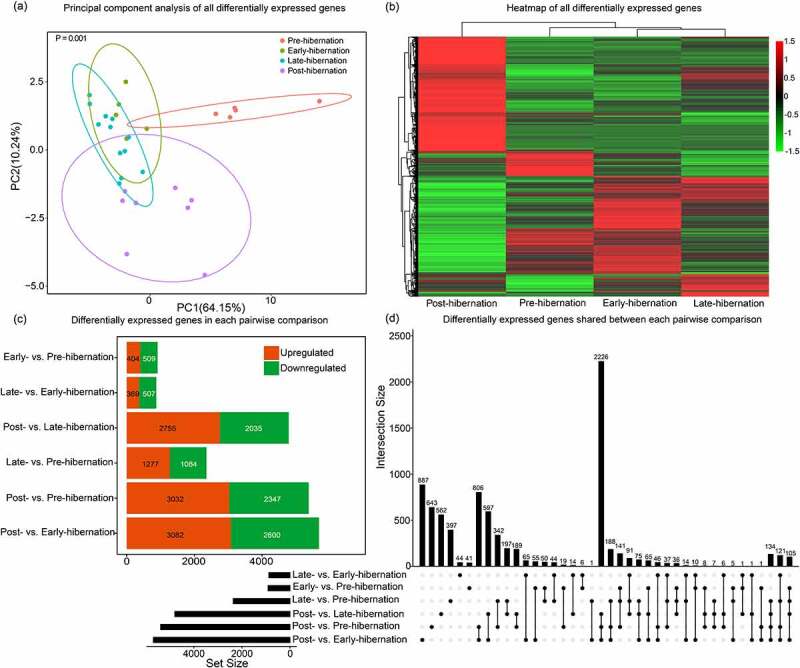
Differential gene-expression analyses in the wing tissues of *R. ferrumequinum* at different physiological stages. (a) Principal component analysis of all DEGs. Percentages of variance explained by PC1 and PC2 are provided, accounting for 64.15% and 10.24%, respectively. *P*-value was obtained by analysis of similarities (ANOSIM). (b) Heatmaps based on all DEGs from six pairwise comparisons of the four physiological stages. Different colours indicate relative expression levels. (c) Number of DEGs identified in each pairwise comparison. The numbers of up- and down-regulated genes are labelled alongside the bar. (d) Upper plot shows the number of DEGs shared between each pairwise comparison.

We conducted six pairwise comparisons for the differential gene-expression analyses across the four physiological stages and identified a total of 8778 DEGs. Our principal component analysis (PCA) based on all DEGs showed four distinct groups, with each corresponding to a distinct physiological stage, and PC1 and PC2 explained 64.15% and 10.24% of the variation, respectively, indicating sizable differences in gene expression among groups (ANOSIM: R = 0.53, *P* = 0.001; Figure 1a). We discerned similar results from the hierarchical clustering (Figure 1b).

When we statistically analysed the number of DEGs, we uncovered 913, 876, 4790, 2361, 5379, and 5682 DEGs in the six groups of pairwise comparisons, respectively, and the total number of DEGs or specific DEGs was the highest in the four groups of comparisons (two post-hibernation vs. hibernation, post- vs. pre-hibernation, and late- vs. pre-hibernation groups). This reflected the largest differences in physiological stages, while the number of up-regulated genes was also greater than the number of down-regulated genes (Figure 1c and 1d). Furthermore, the total number of DEGs was also smaller in the comparison between the two groups, with smaller differences in other physiological stages.

## GO and KEGG pathway enrichment analyses

GO enrichment analysis showed that a total of 261 GO terms were significantly enriched for the six pairwise comparisons of DEGs, of which up- and down-regulated genes were significantly enriched for 180 and 81 GO entries, respectively. We realized that numerous GO terms were enriched by DEGs from the post-hibernation group relative to the other stages and that the up-regulated genes were enriched with more GO terms than were the down-regulated genes. However, fewer terms were significantly enriched for the two hibernation stages vs. pre-hibernation and late-hibernation vs. early-hibernation comparisons (Table 1).

**Table 1.** Number of GO terms significantly enriched for DEGs in six pairwise comparisons.

GO terms number			
	Biological process	Molecular function	Cellular component
	Up/Down	Up/Down	Up/Down
Early – vs. Pre-hibernation	0/5	3/9	1/1
Late- vs. Early-hibernation	0/0	5/5	0/1
Post- vs. Late-hibernation	26/0	38/1	10/0
Late- vs. Pre-hibernation	0/17	3/29	0/15
Post- vs. Pre-hibernation	18/15	52/2	3/0
Post- vs. Early-hibernation	74/0	65/3	4/0

**Figure 2.**
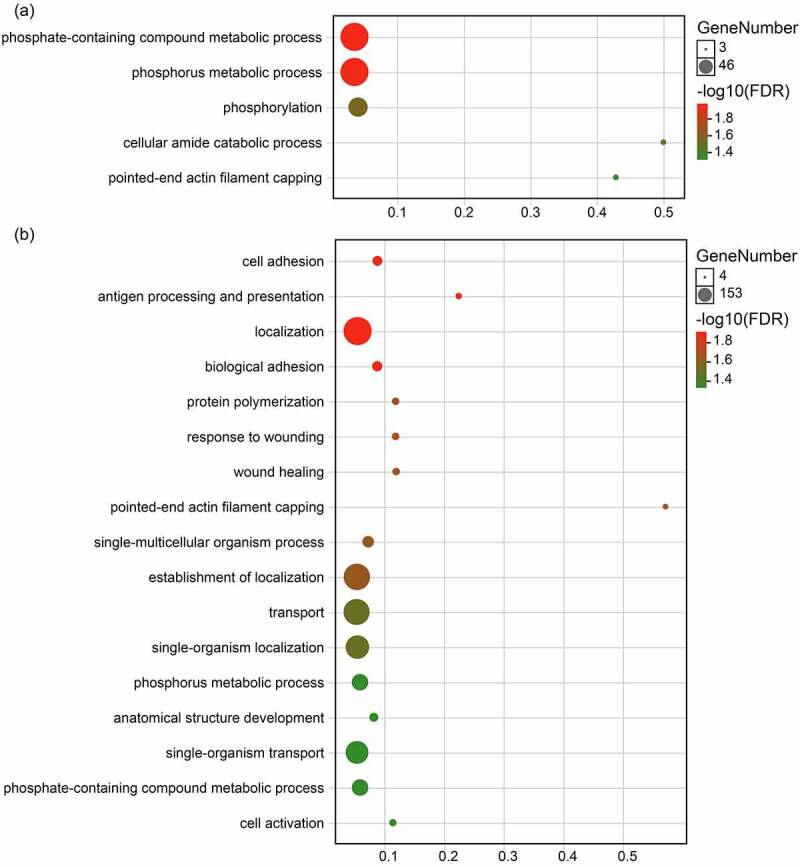
Biological process terms that are significantly enriched for differentially expressed down-regulated genes during hibernation. (a) Early- vs. pre-hibernation and (b) late- vs. pre-hibernation.

The DEGs in the hibernating bats relative to the pre-hibernating bats that were down-regulated in early-hibernation were involved in metabolism (Figure 2a), with many of the most enriched categories involving phosphorus metabolism (e.g. GO: 0006796, phosphate-containing compound metabolic process, *Padj* = 1.87 × 10 − 5). In addition, down-regulated DEGs in late-hibernation were not only enriched in some similar GO terms but were also included in immune response (e.g. antigen processing and presentation), adhesion response (e.g. biological adhesion and wound healing), and transport process (Figure 2b).

**Figure 3.**
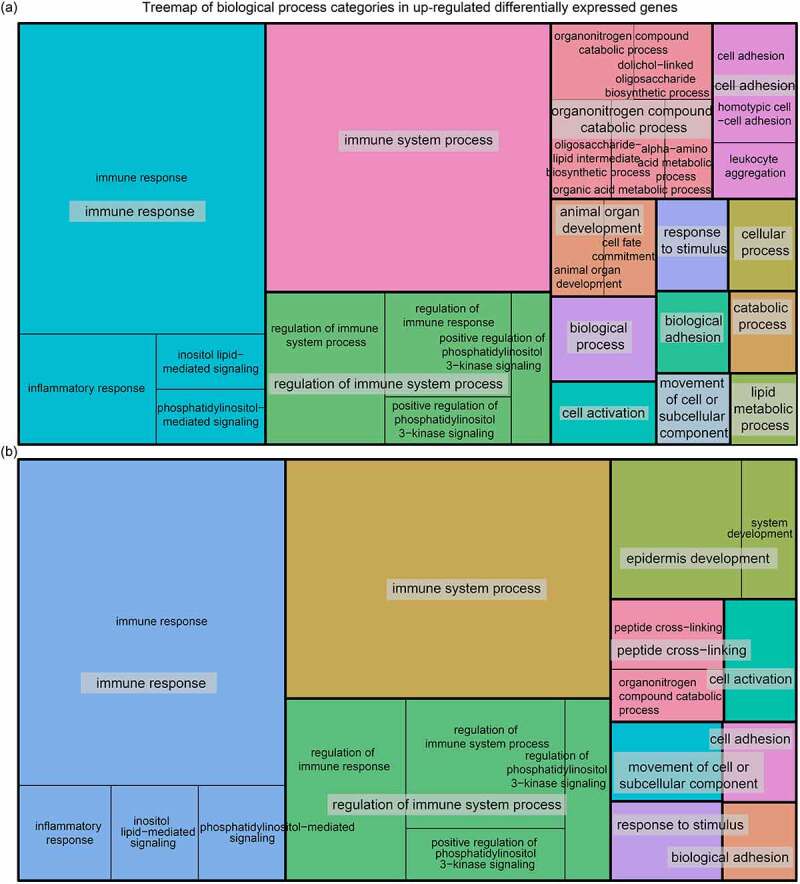
Treemap showing the biological process terms significantly enriched for differentially expressed up-regulated genes in the comparisons of post- vs. early-hibernation (a) and late-hibernation (b). Biological process terms were reduced using Revigo to remove semantic redundancies.

The functional categories of genes in post-hibernation relative to the hibernation groups were enriched in GO terms that included three main categories: immune response, adhesion process, and development process (Figure 3). Specifically, compared to the two stages of hibernation, the up-regulated genes in the post-hibernation group were not only significantly enriched for similar terms (e.g. the three top GO terms with the smallest *Padj* values were immune response, immune system process, and regulation of immune response) but were also significantly enriched for other disparate terms (Table S2). Compared with late-hibernation, the up-regulated genes in the post-hibernation group were significantly enriched for developmentally related terms such as epidermis development and system development, which were not found in the comparison with the early-hibernation group. However, the up-regulated genes in post-hibernation relative to early-hibernation groups were significantly enriched in T-cell related terms such as T-helper 17 type immune response and T cell differentiation, which were not found in the comparison with the late-hibernation group. More importantly, for the late-hibernation vs. early-hibernation comparison, we also discovered that many of the DEGs were involved in cytokine and chemokine activities (Table S3).

**Figure 4.**
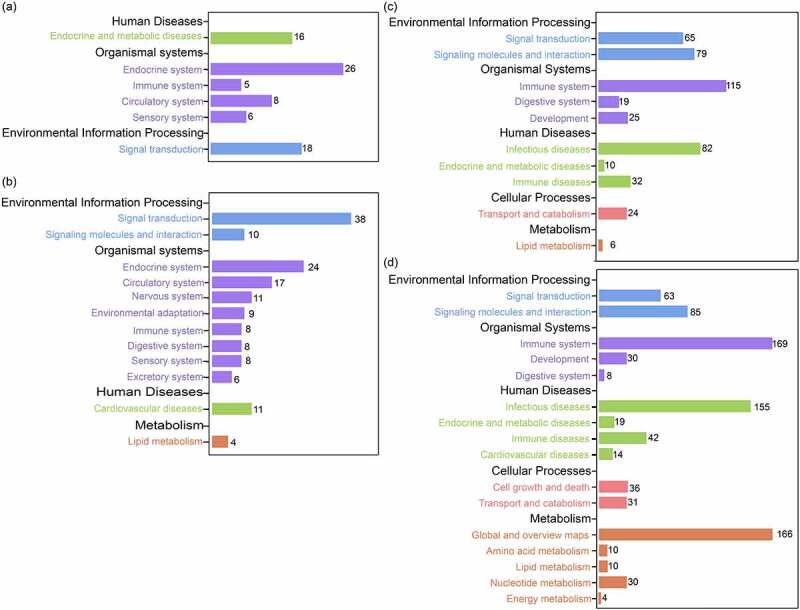
KEGG pathways significantly enriched for DEGs during early-hibernation (a) and late-hibernation (b) vs. pre-hibernation and post-hibernation vs. early-hibernation (c) and late-hibernation (d). (a) and (b) are differentially expressed down-regulated genes, and (c) and (d) are differentially expressed up-regulated genes.

The results of KEGG enrichment analysis revealed that a total of 99 KEGG pathways were significantly enriched in the six pairwise comparisons of DEGs, of which up- and down-regulated genes were significantly enriched for 59 and 40 KEGG pathways, respectively. When we focused on the pathways that were significantly enriched for DEGs during hibernation and post-hibernation (Figure 4), we demonstrated that compared with pre-hibernation, down-regulated genes in the hibernation group were significantly enriched in endocrine system, signal transduction, and immune system pathways. In addition, down-regulated genes were significantly enriched in metabolic pathways between late- vs. pre-hibernation groups, including fatty acid metabolism, biosynthesis of unsaturated fatty acids, and cell adhesion-related pathways, while these pathways were not significantly enriched in the early-hibernation group (Table S4).

When we compared them between the two stages of hibernation, the up-regulated genes in the post-hibernation group were significantly enriched in pathways related to the immune system, signal transduction, and infection diseases. A further analysis of immune system pathways revealed that up-regulated genes were also significantly enriched in the Toll-like receptor signalling pathway, the C-type lectin receptor signalling pathway, and the cytokine signalling pathway (Table 2). In addition, two digestion-related pathways (protein digestion and absorption, and vitamin digestion and absorption) were significantly enriched for up-regulated genes during post-hibernation (Table S5).

**Table 2.** KEGG pathways associated with the immune system of differentially expressed up-regulated genes during post-hibernation compared to hibernation.

Pathway ID	Pathway	Gene Numbers	*P*_*adj*_
**Post- vs. Early-hibernation**			
ko04062	Chemokine signalling pathway	42	3.38E-08
ko04640	Hematopoietic cell lineage	21	2.12E-05
ko04672	Intestinal immune network for IgA production	14	9.12E-04
ko04657	IL-17 signalling pathway	17	1.54E-03
ko04620	Toll-like receptor signalling pathway	17	5.22E-03
ko04662	B cell receptor signalling pathway	16	5.90E-03
ko04610	Complement and coagulation cascades	16	8.50E-03
ko04659	Th17 cell differentiation	19	1.12E-02
ko04650	Natural killer cell mediated cytotoxicity	17	1.12E-02
ko04621	NOD-like receptor signalling pathway	23	1.24E-02
ko04666	Fc gamma R-mediated phagocytosis	17	2.64E-02
ko04623	Cytosolic DNA-sensing pathway	12	4.05E-02
ko04625	C-type lectin receptor signalling pathway	17	4.34E-02
**Post- vs. Late-hibernation**			
ko04062	Chemokine signalling pathway	39	6.18E-09
ko04640	Hematopoietic cell lineage	22	2.10E-07
ko04672	Intestinal immune network for IgA production	13	7.84E-04
ko04610	Complement and coagulation cascades	15	5.56E-03
ko04650	Natural killer cell mediated cytotoxicity	16	7.38E-03
ko04625	C-type lectin receptor signalling pathway	17	1.36E-02
ko04666	Fc gamma R-mediated phagocytosis	15	3.69E-02
ko04620	Toll-like receptor signalling pathway	13	4.10E-02

## Comparison between uninfected bats and bats infected with *Pd* in late hibernation

**Figure 5.**
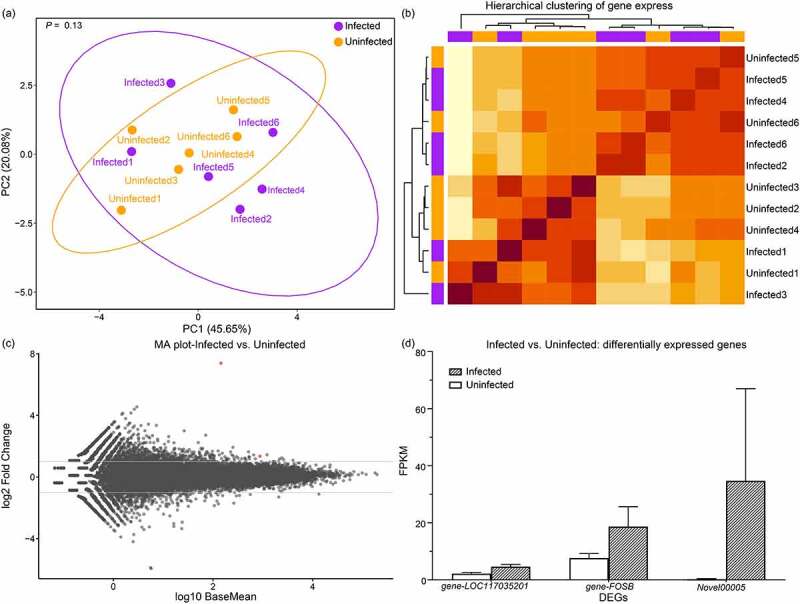
Variation in gene expression between *R. ferrumequinum* specimens that were infected and those that were uninfected with *Pd* in late-hibernation. (a) Principal component analysis showing gene-expression patterns for samples from *Pd*-infected and *Pd*-uninfected bats. The percentages of variance as explained by PC1 and PC2 were 45.65% and 20.08%, respectively. We obtained a *P*-value by ANOSIM. (b) Heatmap of gene expression based on hierarchical clustering analysis using Pearson’s correlation. (c) MA plot showing differential expression between *Pd*-infected bats and *Pd*-uninfected bats. Red points indicate DEGs with a q-value <0.05 and |log_2_ fold-change| > 1 as determined by DESeq2. (d) Bar plot showing the expression levels for three DEGs in infected and uninfected *Pd* bats.

To determine the host response mounted by *R. ferrumequinum* to *Pd* during hibernation, we first compared host gene-expression patterns between infected and uninfected bats. Using PCA, we observed that the first two principal components accounted for approximately 66% of the variation among samples (Figure 5a) and that the gene-expression patterns were similar between the two groups (ANOSIM: R = 0.07, P = 0.18). Hierarchical clustering also consistently showed that infected and uninfected samples were mixed together (Figure 5b). Furthermore, compared with uninfected bats, bats infected with *Pd* showed lower fold-changes in gene expression (Figure 5c). Using DESeq2, we only noted three up-regulated DEGs in *Pd*-infected bats (Figure 5d); of these, the *FOSB* gene (an AP-1 transcription factor) was able to regulate immune-cell activation.

## Discussion

In the present study, we used the RNA-seq approach to examine the bat wing transcriptome based on changes in gene expression in *R. ferrumequinum* that exhibited high resistance to *Pd*. Our data highlighted the dynamic nature of the wing transcriptome, which varied temporally throughout the physiological stages from active to hibernation to active stages. Hibernation is a survival strategy in which animals exhibit reduced body temperature and metabolic rate and experience immune suppression to conserve energy. The reduced metabolic rate of animals during hibernation may lead to changes at the molecular level through regulation of gene expression, just as our results revealed that the genes down-regulated during hibernation compared to pre-hibernation were principally involved in metabolic pathways (Figure 2). Analogous to our findings, the expression of genes involved in oxidoreductase and glycolytic processes (which are also critical to energy metabolism) was shown to be down-regulated in the liver and brain tissues of *R. ferrumequinum* during hibernation [7,47], suggesting a generalized inhibition of metabolism in different tissues of bats during this physiological process.

While DEGs were not enriched for immune-related GO entries during early-hibernation compared to pre-hibernation groups in our study, down-regulated genes in late-hibernation were involved in the immune response, implying a gradual and global down-regulation of host immune system function with increasing hibernation time. It is worth noting, however, that some up-regulated genes in late-hibernation relative to early-hibernation were enriched in GO terms such as chemokine or cytokine activity (Table S3), including *CSF3*, *CCL19*, and *CCL20*. Actually, although hibernation affects host immune system function, the effects of hibernation on intrinsic and specific immune functions during hibernation are divergent due to the exposure of the host to potentially pathogenic infections. Our results therefore provide a new perspective for the study of changes in immune function during hibernation in mammals. On the basis of our results, it is likely that part of the intrinsic immune function may be increased during late-hibernation of the *R. ferrumequinum*, and we speculate that this may be due to cope with potential pathogenic infections. Among these pathogenic infections, an example that is particularly profound for bats is *Pd*, which exhibits seasonal transmission dynamics. Just as we likewise did not detect *Pd* infection in bats during pre-hibernation, but rather detected it during late-hibernation. Fritze et al [48]. similarly found that the whole immune function of highly tolerant *Myotis Myotis* was suppressed during hibernation, but that host innate immunity was enhanced. Thus, the appearance of enhanced intrinsic immune function in the wing tissues of the *R. ferrumequinum* during late-hibernation may be the result of host-pathogen interaction in which bats try to resist *Pd*.

*R.ferrumequinum* also appears to activate an immune reaction to remove a pathogenic infection after bats emerge from hibernation and resume normal activity, with subsequent restoration of all components of the immune system. We demonstrated that up-regulated genes in the post-hibernation group that were involved in C-type lectin receptor signalling pathway and Toll-like receptor signalling pathway included *CLEC7A* and *TLR4* (Table 2) and that these genes were also up-regulated at both the local and systemic levels in response to *Pd* in *M. lucifugus* [26,27]. We thus suggest that after bats emerge from hibernation, responses to pathogenic infection (such as *Pd*) within their bat wing tissue may be mediated by the innate immune system. We also uncovered genes encoding for pro-inflammatory mediators that characterize the innate immune response (including *CSF*, *IL-23A*, and *IL-6*), with increased transcript levels in post-hibernation that potentially mediate the recruitment of monocytes and neutrophils to initiate an adaptive Th17 response and thus provide protection [49]. As a low-level infection is usually contained by an innate immune response, we posit that pathogen clearance may not trigger a robust acute inflammatory response, thus avoiding pathological inflammation.

Although our sampling protocol under native conditions could not eliminate the host’s immune response to other pathogens (including bacteria and viruses), our results clearly demonstrated that these host actions were beneficial in their response to *Pd* infection. We first found that some genes up-regulated in the post-hibernation group relative to the hibernation group were enriched in GO terms associated with skin structural integrity, including cell adhesion, response to stimulus, biological adhesion and animal organ development (Table S2). More importantly, compared with late-hibernating animals with *Pd* infection, the up-regulated genes in the post-hibernation group were also enriched in some GO terms, that play important functions in skin structural re-modelling, such as epidermis development and system development. And this was not observed in the post-hibernation group relative to the early-hibernation group without *Pd* infection. Additionally, KEGG pathway enrichment analysis showed that many up-regulated genes in the post-hibernation group were significantly enriched in the cell adhesion molecules (CAMs) pathway. In fact, the disruption of skin function is considered to be a significant cause of host mortality due to *Pd* infection [50]. Therefore, the increasing expression of genes related to skin structure is significant in host physiological homeostasis and in coping with *Pd* infection after host emergence from hibernation. This is similar to what has been observed in amphibians, where the genes involved in skin structural integrity and re-modelling (e.g. cell-matrix adhesion gene set) were up-regulated in resistant species and down-regulated in susceptible species [24,25,51]. Additionally, *Pd*-produced vitamin B2 and some proteases are the potential virulence factors that erode bat wings, resulting in death [52–54]. Our results also showed that differentially expressed up-regulated genes were associated with vitamin digestion and absorption pathway and protein digestion and absorption pathway, which could potentially facilitate the absorption and digestion of vitamin B2 and of proteases produced by *Pd*. Thus, it may be important for resistant-bat survival to avoid *Pd*-induced damage to wings during hibernation.

The comparison of gene expression between *Pd*-infected and uninfected *R. ferrumequinum* in late-hibernation may have revealed potential physiological tolerance as a host-defence mechanism against pathogens. Previous studies have found a range of DEGs associated with immune responses or other responses in susceptible bats following exposure to *Pd* infection [26,27]. Our results showed that *R. ferrumequinum* appeared to be relatively unresponsive to *Pd* infection, which is different from those susceptible bats hibernating in similar ecological conditions [21]. This result suggests that *R. ferrumeuqinum* exhibits specific patterns that inhibit *Pd* growth. For example, our recent study showed that the skin microbiota of *R. ferrumeuqinum* was enriched in particular taxa with antifungal abilities [33]. Additionally, the bats could eradicate the fungus based on different hibernating behaviours, including different arousing times and hibernating length, which should be tested by behavioural experiments and pathological analysis in future study. Despite this, we reported three genes as exhibiting significantly differential expression in infection with *Pd* (Figure 5). However (and intriguingly), the *FOSB* transcript levels among the three DEGs were also augmented in *M. lucifugus* infected with *Pd* during both torpor and arousal [27]. Investigators reported in a previous study that *FOSB* knockdown significantly reduced the mRNA and protein levels of immune-related chemokines [55], suggesting that bats with high resistance to *Pd* underwent a transformation so as to produce a trade-off between immune response and energy conservation during hibernation, and this then was able to adjust interleukin-17 signalling to prevent tissue damage [56]. *Pd* strains in Asia have coexisted longer with bats than those in North America and Europe, and *Pd*-infected bats during late-hibernation showing an approximately 1000-fold lower fungal load than North American species [57]; this therefore lowers risk following the establishment of equilibrium in host-pathogen interaction [23]. Thus, we hypothesize that this balancing mechanism underlying pathogenic resistance in hibernation may have long been selected by *Pd* pressure, and that it has ultimately been retained throughout co-evolutionary adaptation [58].

## Conclusion

Hibernation allows bats to survive in resource-scarce environments by decreasing their metabolism and immune function, but this also makes them vulnerable to psychrophilic pathogens that include *Pd*. However, we found that a highly resistant species, *R. ferrumequinum*, actually exhibited increased expression of genes related to intrinsic immune responses and avoids acute inflammatory responses during late-hibernation, thus potentially tempering the effects of *Pd* infections. Our data also suggest that improvements in genes related to immune response and skin integrity will facilitate the clearance of *Pd* after the bats emerge from hibernation. The genes identified in the present study may provide inspiration for designing effective interventions in susceptible bats. It also provides a basis for elucidating the mechanisms involved in disease susceptibility, tolerance and resistance to other emerging infectious diseases.

## Supplementary Material

Supplemental Material

## Data Availability

All raw sequencing reads generated were deposited in the NCBI Sequence Read Archive (SRA) under submission accession number SRP337443. The datasets generated and analysed during the current study are available in the Figshare repository (available at: https://figshare.com/s/eb8363e704079baa68fe)
