# Supplementary material for: Temporal dynamics of the bat wing transcriptome: Insight into gene-expression changes that enable protection against pathogen
Source: Virulence. 2023 Jan 4;14(1):2156185. doi: 10.1080/21505594.2022.2156185 (PMC9815227; doi:10.1080/21505594.2022.2156185)
Supplement: Supplemental Material [file KVIR_A_2156185_SM3882.zip › supplementary/Table S1.docx]

Table S1. Statistics of sequencing and mapping of wing membrane samples in different physiological stages of *R. ferrumequinum*.

| **Group** | **Sample number** | **Raw data** | **Clean data** | **Q20** | **Total mapped** | **Uniquely mapped** |
| --- | --- | --- | --- | --- | --- | --- |
| Pre-hibernation | pre-hibernation1 | 46685300 | 45894178 | 0.9706 | 41487596 | 40176392 |
|  | pre-hibernation2 | 39422370 | 38829064 | 0.9728 | 35389374 | 34258632 |
|  | pre-hibernation3 | 44386498 | 43626754 | 0.9706 | 39373930 | 38093484 |
|  | pre-hibernation4 | 44242620 | 43597018 | 0.9718 | 39526548 | 38315058 |
|  | pre-hibernation5 | 47260238 | 46570370 | 0.9725 | 42177652 | 40861396 |
| Early-hibernation | early-hibernation1 | 47591380 | 46928778 | 0.9725 | 42885230 | 41510538 |
|  | early-hibernation2 | 54388106 | 53511426 | 0.9711 | 48513024 | 46981110 |
|  | early-hibernation3 | 52374666 | 51385260 | 0.9702 | 46747208 | 45210224 |
|  | early-hibernation4 | 52520358 | 51665324 | 0.9718 | 47385282 | 45856694 |
|  | early-hibernation5 | 44841654 | 44076922 | 0.9707 | 40428604 | 39125492 |
| Late-hibernation | late-hibernation1 | 52010444 | 51250996 | 0.97 | 46702966 | 45053070 |
|  | late-hibernation2 | 48924028 | 48197714 | 0.971 | 43799382 | 42328286 |
|  | late-hibernation3 | 39411264 | 38859898 | 0.9719 | 35687572 | 34521830 |
|  | late-hibernation4 | 39114158 | 38538500 | 0.9729 | 34573882 | 33492804 |
|  | late-hibernation5 | 42951888 | 42283124 | 0.9729 | 38554290 | 37306004 |
|  | late-hibernation6 | 45792474 | 44709676 | 0.971 | 40841328 | 39504668 |
|  | late-hibernation7 | 50497072 | 49697602 | 0.9725 | 45561148 | 44085444 |
|  | late-hibernation8 | 42932102 | 42247938 | 0.9664 | 38714620 | 37449480 |
|  | late-hibernation9 | 45688156 | 45085814 | 0.9699 | 41193386 | 39732720 |
|  | late-hibernation10 | 42249762 | 41147520 | 0.971 | 37236662 | 36021370 |
|  | late-hibernation11 | 40160584 | 39442548 | 0.9717 | 36024846 | 34875096 |
|  | late-hibernation12 | 43980930 | 41962884 | 0.972 | 37057230 | 35828826 |
| Post-hibernation | post-hibernation1 | 42444902 | 41567056 | 0.9723 | 37008400 | 35573330 |
|  | post-hibernation2 | 48979908 | 48302868 | 0.9715 | 43678622 | 41886572 |
|  | post-hibernation3 | 50477888 | 49466538 | 0.9702 | 44112438 | 42673816 |
|  | post-hibernation4 | 48542190 | 47891750 | 0.9715 | 42748600 | 41150464 |
|  | post-hibernation5 | 45274534 | 44665990 | 0.9715 | 40547548 | 39182740 |
|  | post-hibernation6 | 43992142 | 43583510 | 0.9697 | 39684562 | 38252612 |
|  | post-hibernation7 | 44774050 | 44111204 | 0.9684 | 38392686 | 37062166 |
|  | post-hibernation8 | 46610148 | 45934166 | 0.9727 | 42321868 | 40938680 |
