# Supplementary material for: Temporal dynamics of the bat wing transcriptome: Insight into gene-expression changes that enable protection against pathogen
Source: Virulence. 2023 Jan 4;14(1):2156185. doi: 10.1080/21505594.2022.2156185 (PMC9815227; doi:10.1080/21505594.2022.2156185)
Supplement: Supplemental Material [file KVIR_A_2156185_SM3882.zip › supplementary/Table S2.docx]

Table S2. GO terms significantly enriched for up-regulated genes during Post-hibernation compared to Hibernation.

| **GO ID** | **GO Term** | **GeneRatio** | **BgRatio** | **FDR** |
| --- | --- | --- | --- | --- |
| **Post-hibernation vs. Early-hibernation** | |  |  |  |
| GO:0007159 | leukocyte cell-cell adhesion | 6 | 12 | 0.0493292 |
| GO:0016337 | single organismal cell-cell adhesion | 6 | 12 | 0.0493292 |
| GO:0034109 | homotypic cell-cell adhesion | 6 | 12 | 0.0493292 |
| GO:0042110 | T cell activation | 6 | 12 | 0.0493292 |
| GO:0070486 | leukocyte aggregation | 6 | 12 | 0.0493292 |
| GO:0070489 | T cell aggregation | 6 | 12 | 0.0493292 |
| GO:0071593 | lymphocyte aggregation | 6 | 12 | 0.0493292 |
| GO:0098742 | cell-cell adhesion via plasma-membrane adhesion molecules | 22 | 92 | 0.0493292 |
| GO:1901605 | alpha-amino acid metabolic process | 27 | 121 | 0.0493292 |
| GO:0006629 | lipid metabolic process | 81 | 472 | 0.0493292 |
| GO:0045165 | cell fate commitment | 5 | 8 | 0.039246 |
| GO:0050778 | positive regulation of immune response | 9 | 23 | 0.0371164 |
| GO:0002250 | adaptive immune response | 4 | 5 | 0.0363306 |
| GO:0002285 | lymphocyte activation involved in immune response | 4 | 5 | 0.0363306 |
| GO:0002286 | T cell activation involved in immune response | 4 | 5 | 0.0363306 |
| GO:0002287 | alpha-beta T cell activation involved in immune response | 4 | 5 | 0.0363306 |
| GO:0002292 | T cell differentiation involved in immune response | 4 | 5 | 0.0363306 |
| GO:0002293 | alpha-beta T cell differentiation involved in immune response | 4 | 5 | 0.0363306 |
| GO:0002294 | CD4-positive, alpha-beta T cell differentiation involved in immune response | 4 | 5 | 0.0363306 |
| GO:0002295 | T-helper cell lineage commitment | 4 | 5 | 0.0363306 |
| GO:0002360 | T cell lineage commitment | 4 | 5 | 0.0363306 |
| GO:0002363 | alpha-beta T cell lineage commitment | 4 | 5 | 0.0363306 |
| GO:0002460 | adaptive immune response based on somatic recombination of immune receptors built from immunoglobulin superfamily domains | 4 | 5 | 0.0363306 |
| GO:0002521 | leukocyte differentiation | 4 | 5 | 0.0363306 |
| GO:0030098 | lymphocyte differentiation | 4 | 5 | 0.0363306 |
| GO:0030217 | T cell differentiation | 4 | 5 | 0.0363306 |
| GO:0035710 | CD4-positive, alpha-beta T cell activation | 4 | 5 | 0.0363306 |
| GO:0042093 | T-helper cell differentiation | 4 | 5 | 0.0363306 |
| GO:0043367 | CD4-positive, alpha-beta T cell differentiation | 4 | 5 | 0.0363306 |
| GO:0043368 | positive T cell selection | 4 | 5 | 0.0363306 |
| GO:0043369 | CD4-positive or CD8-positive, alpha-beta T cell lineage commitment | 4 | 5 | 0.0363306 |
| GO:0043373 | CD4-positive, alpha-beta T cell lineage commitment | 4 | 5 | 0.0363306 |
| GO:0045058 | T cell selection | 4 | 5 | 0.0363306 |
| GO:0046631 | alpha-beta T cell activation | 4 | 5 | 0.0363306 |
| GO:0046632 | alpha-beta T cell differentiation | 4 | 5 | 0.0363306 |
| GO:0072538 | T-helper 17 type immune response | 4 | 5 | 0.0363306 |
| GO:0072539 | T-helper 17 cell differentiation | 4 | 5 | 0.0363306 |
| GO:0072540 | T-helper 17 cell lineage commitment | 4 | 5 | 0.0363306 |
| GO:0019882 | antigen processing and presentation | 13 | 40 | 0.0363306 |
| GO:0048513 | animal organ development | 20 | 75 | 0.0363306 |
| GO:0043436 | oxoacid metabolic process | 72 | 398 | 0.0363306 |
| GO:0019752 | carboxylic acid metabolic process | 72 | 396 | 0.0351082 |
| GO:0006928 | movement of cell or subcellular component | 52 | 265 | 0.0347169 |
| GO:0002263 | cell activation involved in immune response | 5 | 7 | 0.0338678 |
| GO:0002366 | leukocyte activation involved in immune response | 5 | 7 | 0.0338678 |
| GO:0002764 | immune response-regulating signaling pathway | 6 | 10 | 0.0338678 |
| GO:0006082 | organic acid metabolic process | 73 | 401 | 0.0338678 |
| GO:0098602 | single organism cell adhesion | 8 | 17 | 0.0338678 |
| GO:0048584 | positive regulation of response to stimulus | 18 | 63 | 0.0338678 |
| GO:0002253 | activation of immune response | 7 | 13 | 0.0331589 |
| GO:0044712 | single-organism catabolic process | 35 | 156 | 0.029799 |
| GO:0006488 | dolichol-linked oligosaccharide biosynthetic process | 4 | 4 | 0.02175 |
| GO:0006490 | oligosaccharide-lipid intermediate biosynthetic process | 4 | 4 | 0.02175 |
| GO:0022610 | biological adhesion | 72 | 384 | 0.02175 |
| GO:0014065 | phosphatidylinositol 3-kinase signaling | 7 | 12 | 0.0214355 |
| GO:0014066 | regulation of phosphatidylinositol 3-kinase signaling | 7 | 12 | 0.0214355 |
| GO:0014068 | positive regulation of phosphatidylinositol 3-kinase signaling | 7 | 12 | 0.0214355 |
| GO:0048015 | phosphatidylinositol-mediated signaling | 7 | 12 | 0.0214355 |
| GO:0048017 | inositol lipid-mediated signaling | 7 | 12 | 0.0214355 |
| GO:0098609 | cell-cell adhesion | 28 | 110 | 0.0200821 |
| GO:1901565 | organonitrogen compound catabolic process | 22 | 77 | 0.0179648 |
| GO:0044763 | single-organism cellular process | 849 | 6240 | 0.0179648 |
| GO:0002684 | positive regulation of immune system process | 12 | 29 | 0.015666 |
| GO:0007155 | cell adhesion | 70 | 360 | 0.015666 |
| GO:0050896 | response to stimulus | 509 | 3557 | 0.0146714 |
| GO:0001775 | cell activation | 31 | 122 | 0.0146714 |
| GO:0044699 | single-organism process | 1066 | 7906 | 0.0033139 |
| GO:0046649 | lymphocyte activation | 11 | 21 | 0.0033139 |
| GO:0045321 | leukocyte activation | 12 | 23 | 0.001677 |
| GO:0050776 | regulation of immune response | 17 | 37 | 0.0002189 |
| GO:0006954 | inflammatory response | 12 | 18 | 6.31E-05 |
| GO:0002682 | regulation of immune system process | 25 | 60 | 9.29E-06 |
| GO:0002376 | immune system process | 104 | 292 | 6.27E-22 |
| GO:0006955 | immune response | 92 | 238 | 6.27E-22 |
| **Post-hibernation vs. Late-hibernation** | |  |  |  |
| GO:0022610 | biological adhesion | 63 | 384 | 0.0425145 |
| GO:0007155 | cell adhesion | 60 | 360 | 0.0393613 |
| GO:1901565 | organonitrogen compound catabolic process | 19 | 77 | 0.0385962 |
| GO:0009888 | tissue development | 27 | 125 | 0.0311485 |
| GO:0048513 | animal organ development | 19 | 75 | 0.0293862 |
| GO:0002764 | immune response-regulating signaling pathway | 6 | 10 | 0.0223777 |
| GO:0019882 | antigen processing and presentation | 13 | 40 | 0.0203484 |
| GO:0048731 | system development | 28 | 125 | 0.0154815 |
| GO:0002684 | positive regulation of immune system process | 11 | 29 | 0.014611 |
| GO:0018149 | peptide cross-linking | 8 | 16 | 0.0131087 |
| GO:0014065 | phosphatidylinositol 3-kinase signaling | 7 | 12 | 0.0107817 |
| GO:0014066 | regulation of phosphatidylinositol 3-kinase signaling | 7 | 12 | 0.0107817 |
| GO:0014068 | positive regulation of phosphatidylinositol 3-kinase signaling | 7 | 12 | 0.0107817 |
| GO:0048015 | phosphatidylinositol-mediated signaling | 7 | 12 | 0.0107817 |
| GO:0048017 | inositol lipid-mediated signaling | 7 | 12 | 0.0107817 |
| GO:0050778 | positive regulation of immune response | 10 | 23 | 0.0107817 |
| GO:0006954 | inflammatory response | 9 | 18 | 0.0081446 |
| GO:0001775 | cell activation | 29 | 122 | 0.0079815 |
| GO:0050896 | response to stimulus | 452 | 3557 | 0.0079815 |
| GO:0006928 | movement of cell or subcellular component | 51 | 265 | 0.0079815 |
| GO:0048584 | positive regulation of response to stimulus | 20 | 63 | 0.0021364 |
| GO:0008544 | epidermis development | 16 | 34 | 4.65E-05 |
| GO:0002682 | regulation of immune system process | 23 | 60 | 1.28E-05 |
| GO:0050776 | regulation of immune response | 18 | 37 | 6.40E-06 |
| GO:0002376 | immune system process | 92 | 292 | 4.03E-19 |
| GO:0006955 | immune response | 85 | 238 | 2.41E-21 |
