# Supplementary material for: Temporal dynamics of the bat wing transcriptome: Insight into gene-expression changes that enable protection against pathogen
Source: Virulence. 2023 Jan 4;14(1):2156185. doi: 10.1080/21505594.2022.2156185 (PMC9815227; doi:10.1080/21505594.2022.2156185)
Supplement: Supplemental Material [file KVIR_A_2156185_SM3882.zip › supplementary/Table S5.docx]

Table S5. KEGG pathways significantly enriched for up-regulated genes during Post-hibernation compared to Hibernation.

| **Pathway ID** | **KEGG_A_class** | **KEGG_B_class** | **Pathway** | **out (720)** | **All (5418)** | **Qvalue** |
| --- | --- | --- | --- | --- | --- | --- |
| **Post- vs. Early-hibernation** | | |  |  |  |  |
| ko04061 | Environmental Information Processing | Signaling molecules and interaction | Viral protein interaction with cytokine and cytokine receptor | 40 | 60 | 3.32E-19 |
| ko04060 | Environmental Information Processing | Signaling molecules and interaction | Cytokine-cytokine receptor interaction | 61 | 152 | 8.14E-15 |
| ko04062 | Organismal Systems | Immune system | Chemokine signaling pathway | 42 | 117 | 3.38E-08 |
| ko04380 | Organismal Systems | Development | Osteoclast differentiation | 30 | 82 | 5.62E-06 |
| ko05323 | Human Diseases | Immune diseases | Rheumatoid arthritis | 25 | 63 | 9.05E-06 |
| ko04668 | Environmental Information Processing | Signal transduction | TNF signaling pathway | 25 | 66 | 2.12E-05 |
| ko04640 | Organismal Systems | Immune system | Hematopoietic cell lineage | 21 | 50 | 2.12E-05 |
| ko05146 | Human Diseases | Infectious diseases | Amoebiasis | 22 | 58 | 7.97E-05 |
| ko05321 | Human Diseases | Immune diseases | Inflammatiory bowel disease (IBD) | 16 | 35 | 1.02E-04 |
| ko05144 | Human Diseases | Infectious diseases | Malaria | 13 | 27 | 4.00E-04 |
| ko04145 | Cellular Processes | Transport and catabolism | Phagosome | 31 | 110 | 7.17E-04 |
| ko04672 | Organismal Systems | Immune system | Intestinal immune network for IgA production | 14 | 33 | 9.12E-04 |
| ko05164 | Human Diseases | Infectious diseases | Influenza A | 30 | 107 | 9.12E-04 |
| ko05152 | Human Diseases | Infectious diseases | Tuberculosis | 31 | 114 | 1.21E-03 |
| ko04630 | Environmental Information Processing | Signal transduction | Jak-STAT signaling pathway | 27 | 95 | 1.49E-03 |
| ko04064 | Environmental Information Processing | Signal transduction | NF-kappa B signaling pathway | 19 | 57 | 1.54E-03 |
| ko04657 | Organismal Systems | Immune system | IL-17 signaling pathway | 17 | 48 | 1.54E-03 |
| ko05150 | Human Diseases | Infectious diseases | Staphylococcus aureus infection | 18 | 56 | 3.78E-03 |
| ko05169 | Human Diseases | Infectious diseases | Epstein-Barr virus infection | 32 | 129 | 4.48E-03 |
| ko04620 | Organismal Systems | Immune system | Toll-like receptor signaling pathway | 17 | 53 | 5.22E-03 |
| ko04662 | Organismal Systems | Immune system | B cell receptor signaling pathway | 16 | 49 | 5.90E-03 |
| ko05168 | Human Diseases | Infectious diseases | Herpes simplex infection | 47 | 218 | 5.90E-03 |
| ko05140 | Human Diseases | Infectious diseases | Leishmaniasis | 19 | 64 | 6.27E-03 |
| ko04610 | Organismal Systems | Immune system | Complement and coagulation cascades | 16 | 51 | 8.50E-03 |
| ko04933 | Human Diseases | Endocrine and metabolic diseases | AGE-RAGE signaling pathway in diabetic complications | 19 | 66 | 8.50E-03 |
| ko05142 | Human Diseases | Infectious diseases | Chagas disease (American trypanosomiasis) | 19 | 66 | 8.50E-03 |
| ko01100 | Metabolism | Global and overview maps | Metabolic pathways | 166 | 1013 | 1.12E-02 |
| ko04659 | Organismal Systems | Immune system | Th17 cell differentiation | 19 | 68 | 1.12E-02 |
| ko04650 | Organismal Systems | Immune system | Natural killer cell mediated cytotoxicity | 17 | 58 | 1.12E-02 |
| ko04514 | Environmental Information Processing | Signaling molecules and interaction | Cell adhesion molecules (CAMs) | 23 | 89 | 1.12E-02 |
| ko00260 | Metabolism | Amino acid metabolism | Glycine, serine and threonine metabolism | 10 | 26 | 1.23E-02 |
| ko04621 | Organismal Systems | Immune system | NOD-like receptor signaling pathway | 23 | 90 | 1.24E-02 |
| ko05332 | Human Diseases | Immune diseases | Graft-versus-host disease | 9 | 23 | 1.77E-02 |
| ko05340 | Human Diseases | Immune diseases | Primary immunodeficiency | 8 | 19 | 1.77E-02 |
| ko04115 | Cellular Processes | Cell growth and death | p53 signaling pathway | 17 | 61 | 1.77E-02 |
| ko05163 | Human Diseases | Infectious diseases | Human cytomegalovirus infection | 33 | 151 | 2.04E-02 |
| ko05133 | Human Diseases | Infectious diseases | Pertussis | 15 | 52 | 2.10E-02 |
| ko05167 | Human Diseases | Infectious diseases | Kaposi sarcoma-associated herpesvirus infection | 28 | 123 | 2.14E-02 |
| ko05162 | Human Diseases | Infectious diseases | Measles | 25 | 106 | 2.14E-02 |
| ko04977 | Organismal Systems | Digestive system | Vitamin digestion and absorption | 8 | 20 | 2.23E-02 |
| ko04666 | Organismal Systems | Immune system | Fc gamma R-mediated phagocytosis | 17 | 64 | 2.64E-02 |
| ko04210 | Cellular Processes | Cell growth and death | Apoptosis | 23 | 97 | 2.71E-02 |
| ko00230 | Metabolism | Nucleotide metabolism | Purine metabolism | 24 | 104 | 3.17E-02 |
| ko05143 | Human Diseases | Infectious diseases | African trypanosomiasis | 8 | 22 | 4.04E-02 |
| ko04623 | Organismal Systems | Immune system | Cytosolic DNA-sensing pathway | 12 | 41 | 4.05E-02 |
| ko05310 | Human Diseases | Immune diseases | Asthma | 7 | 18 | 4.31E-02 |
| ko04625 | Organismal Systems | Immune system | C-type lectin receptor signaling pathway | 17 | 68 | 4.34E-02 |
| ko00240 | Metabolism | Nucleotide metabolism | Pyrimidine metabolism | 14 | 52 | 4.34E-02 |
| ko05416 | Human Diseases | Cardiovascular diseases | Viral myocarditis | 14 | 52 | 4.34E-02 |
| ko00590 | Metabolism | Lipid metabolism | Arachidonic acid metabolism | 10 | 32 | 4.39E-02 |
| ko05145 | Human Diseases | Infectious diseases | Toxoplasmosis | 21 | 91 | 4.57E-02 |
| ko05120 | Human Diseases | Infectious diseases | Epithelial cell signaling in Helicobacter pylori infection | 14 | 53 | 4.85E-02 |
| ko00920 | Metabolism | Energy metabolism | Sulfur metabolism | 4 | 7 | 4.85E-02 |
| **Post- vs. Late-hibernation** | | |  |  |  |  |
| ko04061 | Environmental Information Processing | Signaling molecules and interaction | Viral protein interaction with cytokine and cytokine receptor | 34 | 60 | 1.60E-15 |
| ko04060 | Environmental Information Processing | Signaling molecules and interaction | Cytokine-cytokine receptor interaction | 55 | 152 | 1.33E-14 |
| ko04062 | Organismal Systems | Immune system | Chemokine signaling pathway | 39 | 117 | 6.18E-09 |
| ko04640 | Organismal Systems | Immune system | Hematopoietic cell lineage | 22 | 50 | 2.10E-07 |
| ko04380 | Organismal Systems | Development | Osteoclast differentiation | 25 | 82 | 7.63E-05 |
| ko04630 | Environmental Information Processing | Signal transduction | Jak-STAT signaling pathway | 26 | 95 | 3.67E-04 |
| ko05321 | Human Diseases | Immune diseases | Inflammatiory bowel disease (IBD) | 14 | 35 | 3.93E-04 |
| ko04514 | Environmental Information Processing | Signaling molecules and interaction | Cell adhesion molecules (CAMs) | 24 | 89 | 7.84E-04 |
| ko04672 | Organismal Systems | Immune system | Intestinal immune network for IgA production | 13 | 33 | 7.84E-04 |
| ko05144 | Human Diseases | Infectious diseases | Malaria | 11 | 27 | 2.13E-03 |
| ko05146 | Human Diseases | Infectious diseases | Amoebiasis | 17 | 58 | 3.12E-03 |
| ko05140 | Human Diseases | Infectious diseases | Leishmaniasis | 18 | 64 | 3.26E-03 |
| ko05164 | Human Diseases | Infectious diseases | Influenza A | 25 | 107 | 4.44E-03 |
| ko05150 | Human Diseases | Infectious diseases | Staphylococcus aureus infection | 16 | 56 | 5.42E-03 |
| ko04610 | Organismal Systems | Immune system | Complement and coagulation cascades | 15 | 51 | 5.56E-03 |
| ko04650 | Organismal Systems | Immune system | Natural killer cell mediated cytotoxicity | 16 | 58 | 7.38E-03 |
| ko05332 | Human Diseases | Immune diseases | Graft-versus-host disease | 9 | 23 | 8.39E-03 |
| ko05152 | Human Diseases | Infectious diseases | Tuberculosis | 25 | 114 | 9.06E-03 |
| ko04145 | Cellular Processes | Transport and catabolism | Phagosome | 24 | 110 | 1.19E-02 |
| ko04625 | Organismal Systems | Immune system | C-type lectin receptor signaling pathway | 17 | 68 | 1.36E-02 |
| ko05323 | Human Diseases | Immune diseases | Rheumatoid arthritis | 16 | 63 | 1.52E-02 |
| ko05133 | Human Diseases | Infectious diseases | Pertussis | 14 | 52 | 1.61E-02 |
| ko05330 | Human Diseases | Immune diseases | Allograft rejection | 9 | 26 | 1.75E-02 |
| ko00100 | Metabolism | Lipid metabolism | Steroid biosynthesis | 6 | 13 | 2.03E-02 |
| ko04668 | Environmental Information Processing | Signal transduction | TNF signaling pathway | 16 | 66 | 2.18E-02 |
| ko05310 | Human Diseases | Immune diseases | Asthma | 7 | 18 | 2.45E-02 |
| ko04020 | Environmental Information Processing | Signal transduction | Calcium signaling pathway | 25 | 125 | 2.45E-02 |
| ko05340 | Human Diseases | Immune diseases | Primary immunodeficiency | 7 | 19 | 3.36E-02 |
| ko04666 | Organismal Systems | Immune system | Fc gamma R-mediated phagocytosis | 15 | 64 | 3.69E-02 |
| ko04940 | Human Diseases | Endocrine and metabolic diseases | Type I diabetes mellitus | 10 | 35 | 3.72E-02 |
| ko04620 | Organismal Systems | Immune system | Toll-like receptor signaling pathway | 13 | 53 | 4.10E-02 |
| ko04977 | Organismal Systems | Digestive system | Vitamin digestion and absorption | 7 | 20 | 4.10E-02 |
| ko04974 | Organismal Systems | Digestive system | Protein digestion and absorption | 12 | 48 | 4.66E-02 |
| ko05162 | Human Diseases | Infectious diseases | Measles | 21 | 106 | 4.83E-02 |
