# Supplementary material for: Temporal dynamics of the bat wing transcriptome: Insight into gene-expression changes that enable protection against pathogen
Source: Virulence. 2023 Jan 4;14(1):2156185. doi: 10.1080/21505594.2022.2156185 (PMC9815227; doi:10.1080/21505594.2022.2156185)
Supplement: Supplemental Material [file KVIR_A_2156185_SM3882.zip › supplementary/Table S3.doc]

Table S3. GO terms significantly enriched for differentially expressed up-regulated genes in the comparison of Late- vs. Early-hibernation.

| **GO ID** | **GO Term** | **GeneRatio** | **BgRatio** | **FDR** |
| --- | --- | --- | --- | --- |
| GO:0005126 | cytokine receptor binding | 8 | 124 | 0.0354671 |
| GO:0001664 | G-protein coupled receptor binding | 7 | 78 | 0.0133803 |
| GO:0005125 | cytokine activity | 8 | 97 | 0.0108246 |
| GO:0008009 | chemokine activity | 6 | 40 | 0.0043758 |
| GO:0042379 | chemokine receptor binding | 6 | 40 | 0.0043758 |
