# Supplementary material for: Temporal dynamics of the bat wing transcriptome: Insight into gene-expression changes that enable protection against pathogen
Source: Virulence. 2023 Jan 4;14(1):2156185. doi: 10.1080/21505594.2022.2156185 (PMC9815227; doi:10.1080/21505594.2022.2156185)
Supplement: Supplemental Material [file KVIR_A_2156185_SM3882.zip › supplementary/Table S4.doc]

Table S4. KEGG pathways significantly enriched for down-regulated genes during Hibernation compared to Pre-hibernation.

| **Pathway ID** | **KEGG_A_class** | **KEGG_B_class** | **Pathway** | **out (104)** | **All (5418)** | **Qvalue** |
| --- | --- | --- | --- | --- | --- | --- |
| **Early- vs. Pre-hibernation** | | |  |  |  |  |
| ko04931 | Human Diseases | Endocrine and metabolic diseases | Insulin resistance | 9 | 62 | 0.000487 |
| ko04911 | Organismal Systems | Endocrine system | Insulin secretion | 7 | 45 | 0.001506 |
| ko04725 | Organismal Systems | Nervous system | Cholinergic synapse | 8 | 62 | 0.001506 |
| ko04922 | Organismal Systems | Endocrine system | Glucagon signaling pathway | 8 | 74 | 0.004162 |
| ko04020 | Environmental Information Processing | Signal transduction | Calcium signaling pathway | 10 | 125 | 0.005564 |
| ko05410 | Human Diseases | Cardiovascular diseases | Hypertrophic cardiomyopathy (HCM) | 7 | 64 | 0.007406 |
| ko05414 | Human Diseases | Cardiovascular diseases | Dilated cardiomyopathy (DCM) | 7 | 67 | 0.008478 |
| ko04261 | Organismal Systems | Circulatory system | Adrenergic signaling in cardiomyocytes | 8 | 101 | 0.016857 |
| ko04152 | Environmental Information Processing | Signal transduction | AMPK signaling pathway | 7 | 78 | 0.016857 |
| ko04918 | Organismal Systems | Endocrine system | Thyroid hormone synthesis | 5 | 39 | 0.018165 |
| ko04925 | Organismal Systems | Endocrine system | Aldosterone synthesis and secretion | 6 | 61 | 0.020808 |
| ko04022 | Environmental Information Processing | Signal transduction | cGMP - PKG signaling pathway | 8 | 117 | 0.031147 |
| ko04750 | Organismal Systems | Sensory system | Inflammatory mediator regulation of TRP channels | 6 | 68 | 0.031147 |
| ko04610 | Organismal Systems | Immune system | Complement and coagulation cascades | 5 | 51 | 0.044131 |
| **Late- vs. Pre-hibernation** | | |  |  |  |  |
| ko04020 | Environmental Information Processing | Signal transduction | Calcium signaling pathway | 17 | 125 | 0.000517 |
| ko04725 | Organismal Systems | Nervous system | Cholinergic synapse | 11 | 62 | 0.001147 |
| ko04261 | Organismal Systems | Circulatory system | Adrenergic signaling in cardiomyocytes | 14 | 101 | 0.001147 |
| ko05414 | Human Diseases | Cardiovascular diseases | Dilated cardiomyopathy (DCM) | 11 | 67 | 0.001484 |
| ko05410 | Human Diseases | Cardiovascular diseases | Hypertrophic cardiomyopathy (HCM) | 10 | 64 | 0.004368 |
| ko03320 | Organismal Systems | Endocrine system | PPAR signaling pathway | 8 | 43 | 0.004648 |
| ko04260 | Organismal Systems | Circulatory system | Cardiac muscle contraction | 10 | 67 | 0.004648 |
| ko04911 | Organismal Systems | Endocrine system | Insulin secretion | 8 | 45 | 0.005629 |
| ko04022 | Environmental Information Processing | Signal transduction | cGMP - PKG signaling pathway | 13 | 117 | 0.007561 |
| ko04922 | Organismal Systems | Endocrine system | Glucagon signaling pathway | 10 | 74 | 0.007561 |
| ko04658 | Organismal Systems | Immune system | Th1 and Th2 cell differentiation | 8 | 52 | 0.011502 |
| ko04713 | Organismal Systems | Environmental adaptation | Circadian entrainment | 9 | 66 | 0.011848 |
| ko04971 | Organismal Systems | Digestive system | Gastric acid secretion | 8 | 55 | 0.01435 |
| ko04514 | Environmental Information Processing | Signaling molecules and interaction | Cell adhesion molecules (CAMs) | 10 | 89 | 0.023426 |
| ko01212 | Metabolism | Global and overview maps | Fatty acid metabolism | 6 | 35 | 0.023426 |
| ko04925 | Organismal Systems | Endocrine system | Aldosterone synthesis and secretion | 8 | 61 | 0.023426 |
| ko04931 | Human Diseases | Endocrine and metabolic diseases | Insulin resistance | 8 | 62 | 0.024544 |
| ko04152 | Environmental Information Processing | Signal transduction | AMPK signaling pathway | 9 | 78 | 0.026774 |
| ko04024 | Environmental Information Processing | Signal transduction | cAMP signaling pathway | 13 | 144 | 0.027154 |
| ko04918 | Organismal Systems | Endocrine system | Thyroid hormone synthesis | 6 | 39 | 0.031591 |
| ko01040 | Metabolism | Lipid metabolism | Biosynthesis of unsaturated fatty acids | 4 | 17 | 0.031591 |
| ko05412 | Human Diseases | Cardiovascular diseases | Arrhythmogenic right ventricular cardiomyopathy (ARVC) | 7 | 53 | 0.031591 |
| ko04961 | Organismal Systems | Excretory system | Endocrine and other factor-regulated calcium reabsorption | 6 | 40 | 0.031591 |
| ko04750 | Organismal Systems | Sensory system | Inflammatory mediator regulation of TRP channels | 8 | 68 | 0.031591 |
